# The role of ultrasound in diagnosing 2mm medullary thyroid carcinoma with cervical lymph node metastasis: a case report

**DOI:** 10.3389/fonc.2025.1588295

**Published:** 2025-09-26

**Authors:** Qinyun Wan, Li Sun, Lu Gan, Jianxue Liu

**Affiliations:** ^1^ Department of Ultrasound Medicine, Baoji Central Hospital, Baoji, China; ^2^ Department of Pathology, Baoji Central Hospital, Baoji, China

**Keywords:** thyroid medullary carcinoma, lymph node metastasis, high-frequency ultrasound, fine needle aspiration, cytology

## Abstract

**Background:**

Medullary thyroid carcinoma (MTC) is a rare, aggressive thyroid cancer, comprising 5%-10% of cases, often leading to cervical lymph node metastasis.

**Case presentation:**

We report a 49-year-old woman with a 2 mm micro-nodule in the left thyroid lobe and two suspicious lymph nodes in the neck. Ultrasound-guided fine needle aspiration indicated MTC, confirmed by immunohistochemical analysis. Following total thyroidectomy and left cervical lymphadenectomy, two of 26 lymph nodes showed metastasis. The cancer was 2 mm, with no capsular invasion, and positive markers for MTC were noted. This case emphasizes the importance of early MTC detection in micro-nodules and thorough lymph node evaluation.

**Conclusion:**

Even a 2 mm MTC has the potential to metastasize lateral cervical lymph nodes. This emphasizes the necessity of meticulous ultrasound evaluation by experienced clinicians to detect abnormal lymph nodes and inform clinical management. Interventional ultrasound plays a crucial role in both diagnosing MTC micro-nodules and identifying associated lymph node metastases.

## 1 Introduction

Medullary thyroid carcinoma (MTC) is an aggressive and uncommon thyroid cancer, accounting for 5-10% of all thyroid malignancies. It arises from parafollicular C cells, which produce calcitonin (CT), and is characterized by its unique biological behavior distinct from differentiated thyroid cancer. The prognosis for MTC is generally poor because it tends to spread early through the lymphatic system and has limited responsiveness to conventional therapies, including radiotherapy and chemotherapy (1). This malignancy represents a significant clinical challenge, particularly as it often presents with indolent clinical features that may lead to delayed diagnosis and treatment (2).

Current diagnostic methods, such as genetic screening for Rearranged during Transfection (RET) proto-oncogene mutations and calcitonin level measurements, are essential for identifying hereditary forms of MTC and for early detection in at-risk populations. Genetic testing enables facilitate timely intervention, thus significantly improving patient outcomes (3). However, the role of imaging techniques in the early detection of MTC remains underexplored, particularly for small nodules less than 5 mm, which may be missed on conventional modalities (4).

While advances in imaging techniques, detecting small, clinically significant thyroid nodules, particularly for small lesions, remains challenging. Ultrasound-guided fine-needle aspiration (FNA) is now the cornerstone for assessing thyroid nodules. Evidence demonstrates that ultrasonography enhances the detection of microcarcinomas. It also increases the diagnostic yield for MTC, especially when used alongside cytological and immunohistochemical analysis (3). Nevertheless, there remains a gap in the literature regarding the specific ultrasound characteristics of small MTC nodules (<10 mm) and their associated lymph node metastases.

We report a case of a 2 mm MTC confirmed by pathology, with central and lateral neck lymph node metastases. To our knowledge, no previous cases have documented ultrasound-guided fine needle aspiration cytology suspicious for MTC, which was ultimately confirmed by histopathology after surgery, with MTC measuring less than 2 mm accompanied by cervical lymph node metastasis. This case highlights that early detection of microcarcinomas could lead to timely surgical intervention, thereby improving patient prognosis and potentially reducing the economic burden of advanced disease management.

## 2 Case presentation

### 2.1 Patient history

In October 2019, a 49-year-old female patient underwent a health screening at another hospital. An ultrasound examination revealed a solitary nodule on the left thyroid (size unknown). Serum tests indicated hypothyroidism, prompting initiation of levothyroxine treatment at 75 μg/day. On April 8, 2020, a physical examination at our hospital showed a normal thyroid with appropriate size, soft texture, and no tenderness. Her heart rate was 88 beats per minute. The patient had no family history of thyroid cancer.

### 2.2 Ultrasound examination

The patient underwent two preoperative thyroid ultrasound examinations at our institution. The initial examination used a Samsung XR80A ultrasound system with a high-frequency linear probe (L3-12A; frequency range: 3.0–12.0 MHz), while the follow-up examination employed a Supersonic Imaging Aixplorer system with a high-frequency linear probe (SL15-4; frequency range: 4.0–15.0 MHz). Prior to each scan, the patient was positioned supine with full neck exposure for comprehensive evaluation of the thyroid gland and cervical lymph nodes.

The patient’s initial thyroid ultrasound examination was performed by a junior physician at our hospital on April 8, 2020, revealing a thyroid of normal size and contour with homogeneous echogenicity. A 0.24×0.27×0.22 cm nodule was identified in the left lobe demonstrating an aspect ratio >1, central parenchymal location without capsular contact, markedly hypoechoic echogenicity, unclear margins, and solid composition without calcifications. Color Doppler imaging showed no intranodular vascular flow. According to the Thyroid Imaging Reporting and Data System (TI-RADS) scoring system published by the American College of Radiology (ACR), the nodule was scored 8 points and classified as TR5. Given the maximal diameter of 0.27 cm, clinical follow-up was recommended in accordance with ACR management guidelines.

**Figure 1:**
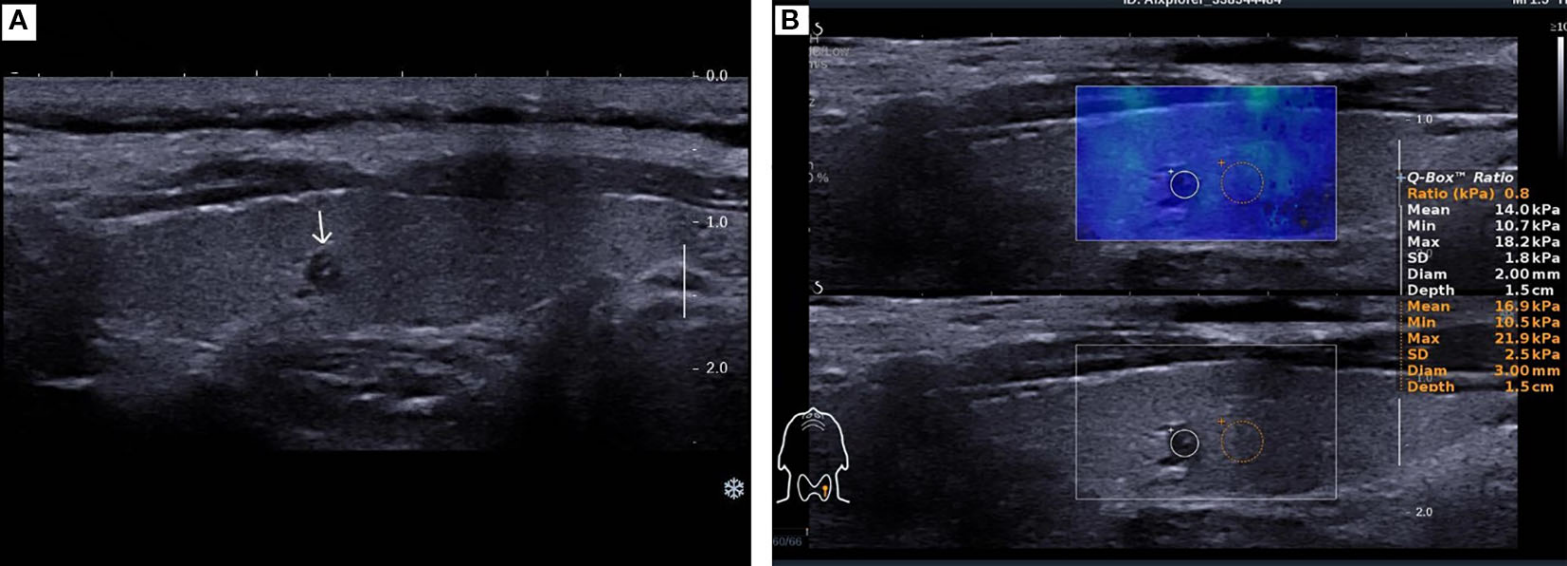
A 49-year-old female patient was found to have a 2 mm nodule in the left lobe of the thyroid. **(A)**. Two-dimensional ultrasound image of the thyroid micro-nodule. **(B)**. Elastography image of the thyroid micro-nodule, indicating an elasticity ratio of 0.8 relative to the surrounding thyroid tissue.

**Figure 2:**
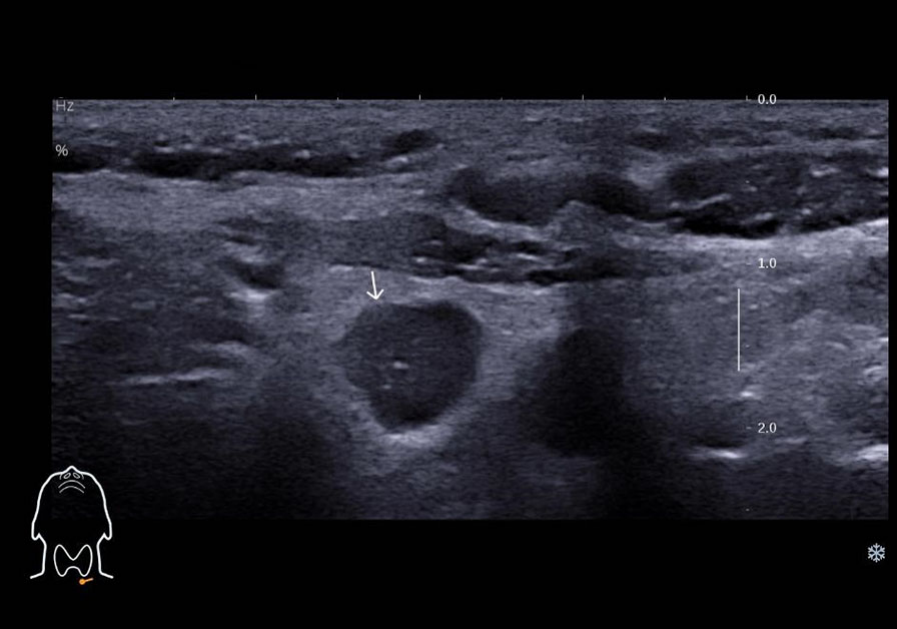
Ultrasound image depicting lymph node metastasis of medullary thyroid carcinoma in cervical region VI, demonstrating internal fine calcifications.

**Figure 3:**
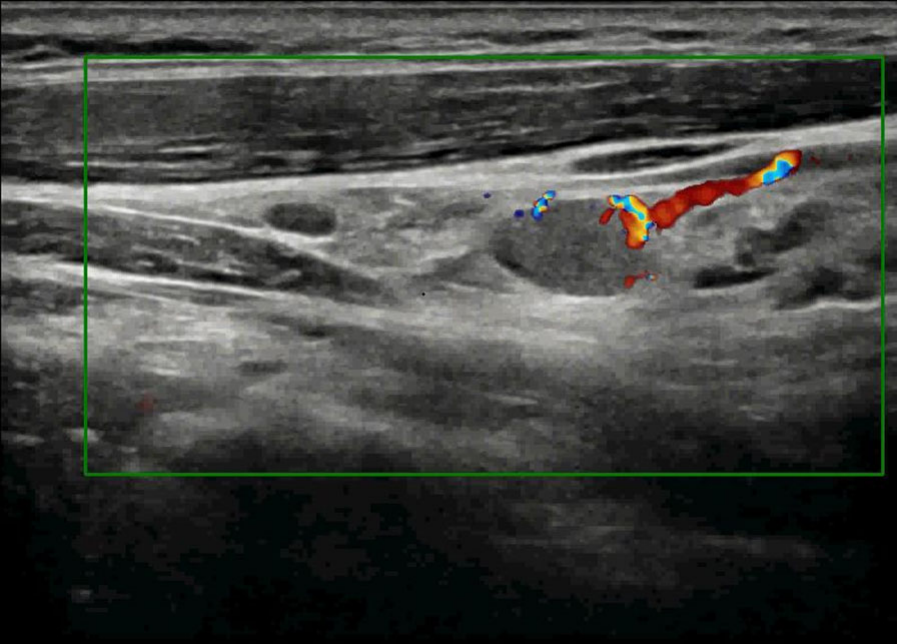
Color Doppler ultrasound demonstrates metastatic involvement in cervical region III, revealing sparse peripheral blood flow signals within the lesion.

The patient underwent a follow-up thyroid ultrasound with a senior physician on September 23, 2020, revealing a left thyroid lobe nodule measuring 0.23×0.26×0.22 cm without significant interval change compared to the prior study six months earlier; However, the senior physician observed small hyperechoic foci within the thyroid nodule, a critical finding that upgraded its ACR TI-RADS classification to 11 points (TR5; **Figures 1A, B**). Concurrently, two abnormal lymph nodes were identified in the left neck at Levels VI and III, respectively. The Level VI node (1.0×0.8×0.9 cm) demonstrated an irregular contour with indistinct fatty hilum, further revealing cortical thickening, complete medullary architecture loss, internal punctate calcifications, and sparse vascular flow (**Figure 2**). In direct comparison, the Level III node (1.0×0.5×0.6 cm) maintained a regular shape but similarly exhibited an indistinct hilum, cortical thickening, and effaced medullary architecture, though distinctively lacking calcifications or cystic changes while showing sparse internal vascularity (**Figure 3**). Given the nodule’s escalated TI-RADS classification (TR5) and radiographic evidence suggestive of lymph node metastasis, ultrasound-guided fine-needle aspiration cytology was performed.

### 2.3 Fine needle aspiration biopsy

On October 8, 2020, the patient underwent ultrasound-guided FNA of the left thyroid nodule and left neck lymph nodes. The aspirated samples were preserved in liquid-based cytology vials and vortexed after differential centrifugation. Following 30 seconds of vortexing, 2 ml of buffer was added for further mixing. Using the LBP liquid-based cytology system, slides were prepared with 200 μl of sediment per slide and subsequently stained with Hematoxylin and Eosin (H&E) for microscopic evaluation.

The fine-needle aspiration specimen from the left thyroid nodule was deemed adequate for diagnosis. The sample revealed scattered atypical epithelial cells with irregular contours and pleomorphic sizes. These cells exhibited abundant eosinophilic cytoplasm and eccentrically positioned nuclei of variable dimensions, including some markedly enlarged forms. The nuclei demonstrated thickened membranes, prominent nucleoli, and coarsely granular chromatin. The cytomorphological features were consistent with thyroid carcinoma, though histological subtyping requires further tissue confirmation. The immunohistochemical results and special stains, including acidophilic red, Thyroid transcription factor-1 (TTF-1), Galectin-3, Thyroglobulin (Tg), cytokeratin (CK) 19, and CT, were inconclusive. This was due to the disappearance of target cells during processing.

**Figure 4:**
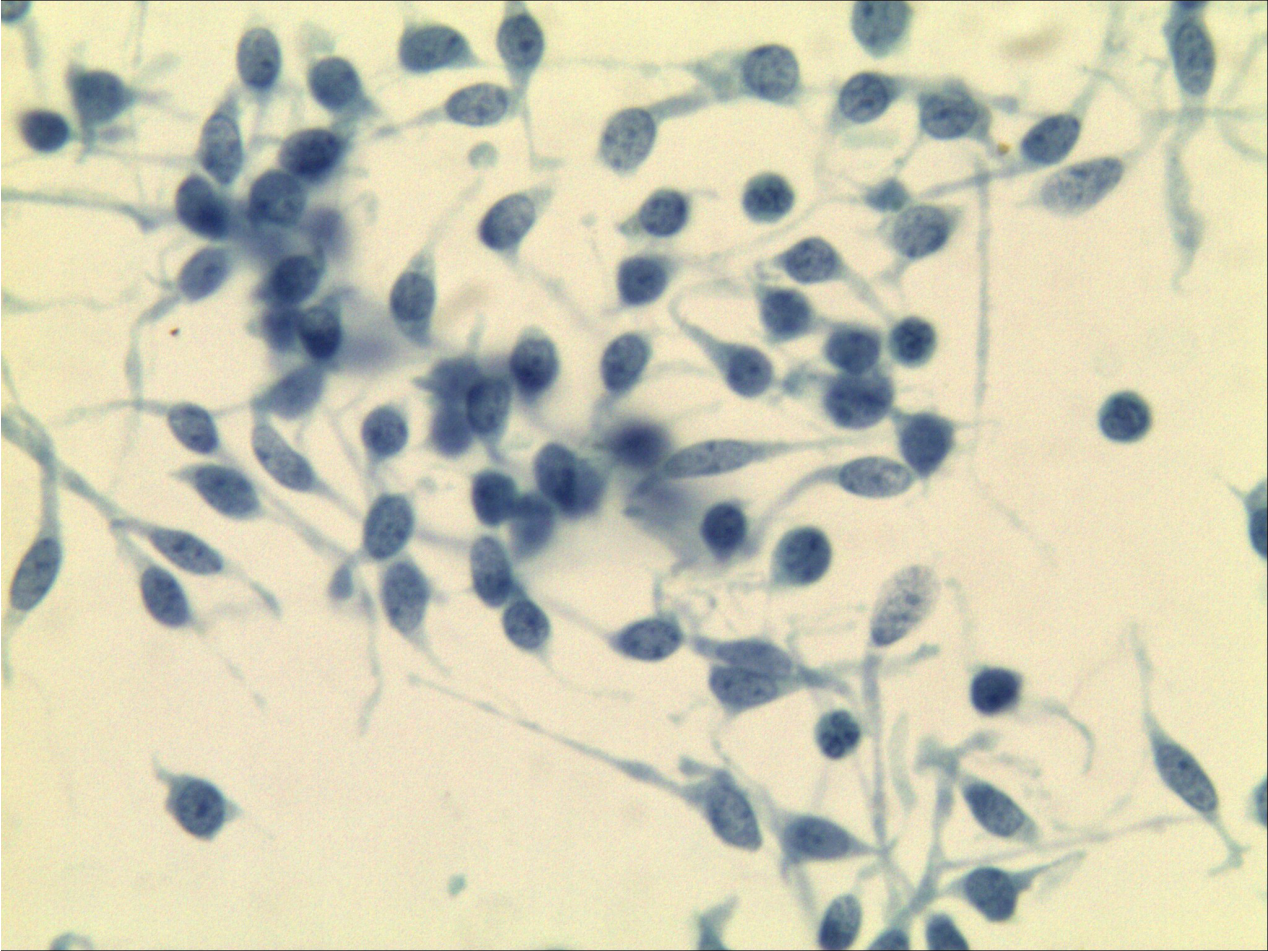
Lymph node cytopathology images. The morphology combined with immunohistochemical results [TTF-1 (++), CT (+), Syn (+++), CK (+++)] supported a neuroendocrine tumor, medullary thyroid carcinoma could be suspected (Papanicolaou staining ×400).

The specimen from the left neck lymph node was satisfactory. The specimen contained numerous small, lymphocyte-like cells, which were either scattered or clustered and exhibited fragile to minimal cytoplasm. Some tumor cells appeared spindle-shaped, with generally round or oval nuclei showing mild atypia. The nuclear chromatin was fine-grained, with a salt-and-pepper pattern, and eosinophilic material deposits were observed within and between cell clusters. Immunohistochemistry and special staining were performed on tissue fragments obtained through fine-needle aspiration, yielding the following results: TTF-1 (++), CT (+), Synaptophysin (Syn) (+++), and CK (+++). The morphological features, combined with the immunohistochemical profile, supported the diagnosis of a neuroendocrine tumor. In conjunction with clinical findings—a thyroid mass and enlarged neck lymph nodes—MTC could not be excluded (**Figure 4**).

The hospital experienced a temporary shortage of specific reagents required for serum calcitonin measurement in 2020. However, reagents for measuring CT levels in aspirated samples remained available and operational. This enabled preoperative assessment of CT levels in aspirated samples, while serum measurement was suspended due to the reagent shortage.

### 2.4 Thyroidectomy and neck lymph node dissection

On October 21, 2020, the patient underwent total thyroidectomy with neck lymph node dissection under general anesthesia, during which intraoperative frozen section pathology revealed poorly differentiated carcinoma suggestive of medullary thyroid carcinoma. Subsequent examination of postoperative paraffin sections demonstrated cancer tissue arranged in solid sheets, nests, trabeculae, and glandular patterns exhibiting infiltrative growth, along with stromal fibrous hyperplasia and focal amyloid deposits.

**Figure 5:**
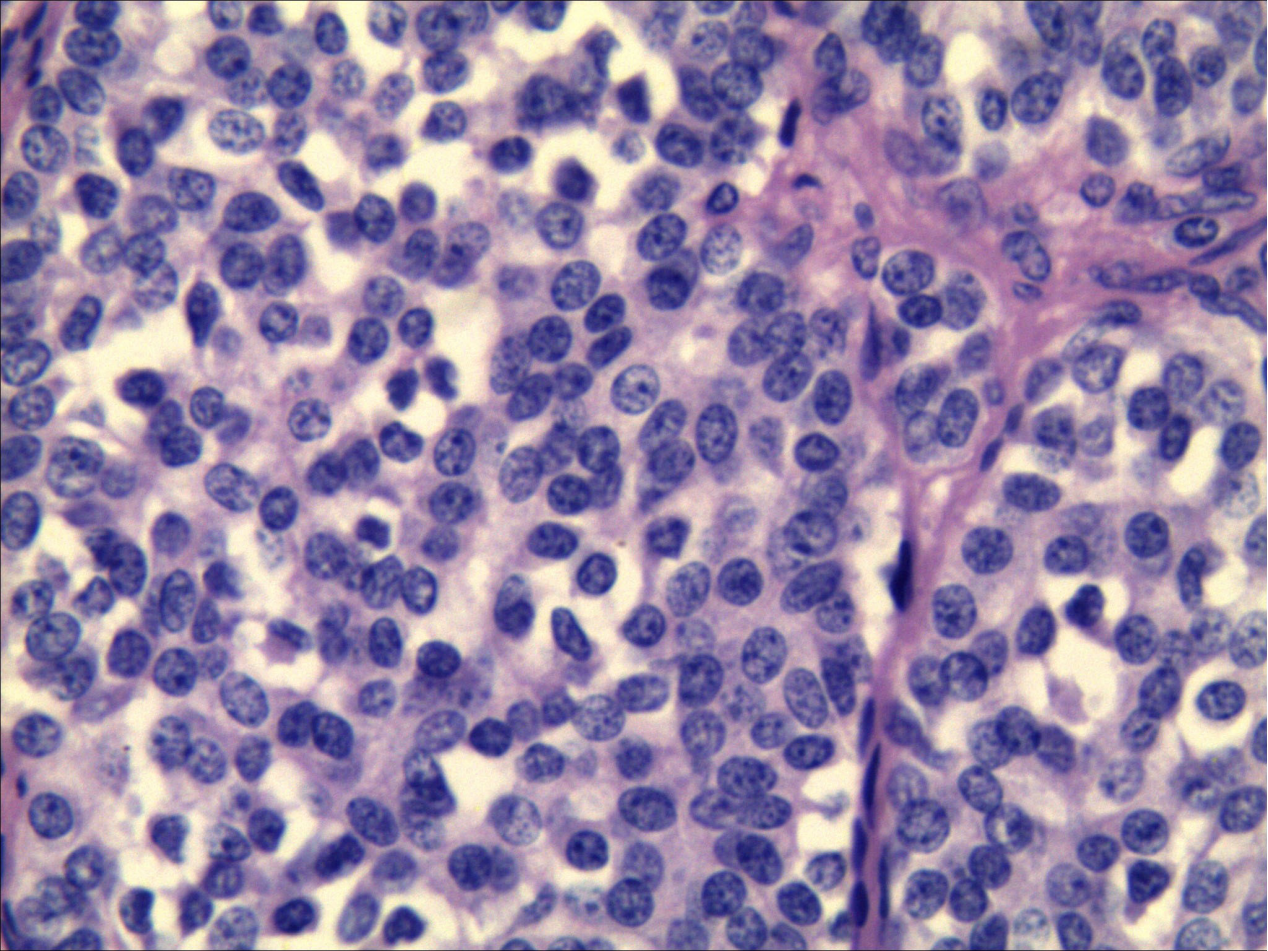
Pathological results after surgical resection of thyroid nodules. Morphology combined with immunohistochemistry confirmed medullary thyroid carcinoma. [HE staining ×400, CT (+), CEA (+), Syn (+), CgA (+), TTF-1 (+), Ki-67 (10%), CK (+)].

The tumor cells displayed round, polygonal, or plump spindle morphology containing moderate amphophilic cytoplasm, with round-to-oval nuclei showing salt-and-pepper chromatin and rare mitoses. Immunohistochemical analysis confirmed CT (+), Carcinoembryonic antigen (CEA) (+), Syn (+), CgA(+), TTF-1(+), Ki-67(10%), and CK (+) expression, while special stains revealed Congo red (methanol method) (+) and acid-fast red (+) reactivity. These morphological and immunohistochemical findings collectively confirmed medullary thyroid carcinoma (**Figure 5**), with the largest tumor focus measuring 0.2 cm without capsular invasion; adjacent thyroid tissue exhibited nodular goiter.

Lymph node dissection retrieved 26 lymph nodes in total, including 3 from the central compartment (Level VI; 1 with metastasis) and 23 from Levels III and IV (1 with metastasis).

### 2.5 Follow-up

Following thyroidectomy, received daily oral levothyroxine sodium supplementation (75 µg). Thyroid function tests are currently within normal ranges. During June 2024 Follow-up neck ultrasound demonstrated no abnormalities, with serum CT level measuring 10 pg/ml.

## 3 Discussion

MTC exhibits distinct biological behaviors and is associated with variable clinical outcomes. Although clinically rare, it accounts for to 8% to 13% of thyroid cancer-related deaths, with a 10-year survival rates ranging from 69% to 89% (5). Studies have reported an increasing incidence of MTC, a heightened propensity for distant metastasis, and an overall poor prognosis. Cervical lymph node metastasis rates occurs in up to 70% of cases, with larger the tumor size correlating strongly with increased nodal involvement (6–9).Previous literature has emphasized the critical role of early diagnosis in improving patient prognosis, yet the ultrasound characteristics of microcarcinomas associated with MTC remain underexplored. By documenting a case of MTC identified through ultrasound and correlating it with lymph node involvement, this research highlights the need for comprehensive examinations of the neck when suspicious nodules are detected. The findings will contribute to filling the existing gap in knowledge regarding the early detection and management of this challenging malignancy (10).

According to previous literature, differences in sonographic features exist between micro-MTC (≤1 cm) and typical MTC (>1 cm). Typical MTC often presents as isoechoic, with regular margins, an aspect ratio <1, and abundant vascularity. It is also more prone to cystic degeneration, resembling features of benign lesions. In contrast, micro-MTC commonly appears hypoechoic with irregular margins and reduced vascularity, mimicking the imaging characteristics of PTC. However, an aspect ratio ≤1 is more frequently observed in micro-MTC than in PTC. In this case, the lesion demonstrated hypoechogenicity, ill-defined margins, an aspect ratio >1, and microcalcifications—features highly consistent with typical PTC rather than MTC (11–13).

Tumor size, multifocality, bilaterality, extrathyroidal extension, and capsular invasion are established risk factors for cervical lymph node metastasis in MTC (14, 15). Notably, this case demonstrated nodal metastasis despite being a unifocal microcarcinoma (0.2 cm) with no capsular proximity. The finding underscores that even micro-nodules may harbor metastatic potential, evidenced by two pathologically confirmed metastatic nodes in the left cervical compartment. Patients presenting with such small nodules may exhibit distinct clinical characteristics compared to the general thyroid cancer population. Heightened awareness of sonographic features specific to micro-MTC could improve early detection rates. Age and gender significantly influence MTC incidences (2),with suggesting these demographic factors should inform screening protocols. Advanced ultrasonography techniques offer particular promises for enhancing early detection, potentially enabling intervention at more treatable disease stages and thereby improving prognostic outcomes.

The pathological examination revealed a tumor measuring 2 mm in maximum diameter with positive immunohistochemical staining for CT and CEA, confirming MTC. These specific pathological features are critical for diagnostic confirmation and clinical management. The prognostic significance of immunohistochemical markers is well-established, with certain markers indicating a more aggressive disease course. Immunohistochemical analysis reliably differentiates MTC from other thyroid neoplasms and enables personalized treatment strategies based on the tumor’s biological behavior.

The total thyroidectomy and lymph node dissection demonstrates the critical importance of early surgical intervention for optimizing outcomes in MTC patients. The intraoperative identification of metastatic lymph nodes confirms the aggressive biology of this malignancy, necessitating proactive surgical management regardless of primary tumor size. This approach aligns with contemporary guidelines recommending comprehensive resection for MTC, particularly with suspected nodal involvement. Our findings reflect current management paradigms where early surgery correlates with improved survival. Future research should investigate optimal surgical timing/extent and adjuvant therapy integration to further enhance prognostic outcomes.

This case report presents several limitations, including the absence of preoperative testing for the patient’s calcitonin levels. Furthermore, the status of the RET mutation could not be assessed postoperatively.

In conclusion, this case report emphasizes the potential of ultrasound in the early diagnosis of thyroid tumor, particularly in identifying nodules around 2 millimeters and associated lymph node metastases. It highlights the necessity of a thorough assessment of the cervical lymphatic structures for suspicious thyroid nodules. Senior physicians using high-frequency ultrasound are beneficial for detecting lymph node metastases of small thyroid cancers, and ultrasound-guided FNA is useful for the diagnosis of small MTC.

## Data Availability

The original contributions presented in the study are included in the article/supplementary material. Further inquiries can be directed to the corresponding author.
